# Prescribing exercise as a treatment for depression

**DOI:** 10.1192/j.eurpsy.2021.904

**Published:** 2021-08-13

**Authors:** F. Pereira, P. Martins, J. Barros

**Affiliations:** 1 Departamento De Psiquiatria E Saúde Mental, Unidade Local de Saúde do Nordeste, Porto, Portugal; 2 Departamento De Psiquiatria E Saúde Mental, Unidade Local de Saúde do Nordeste, Maia, Pedrouços, Portugal

**Keywords:** Physical Activity, Exercise

## Abstract

**Introduction:**

Depression is a heterogeneous syndrome linked to significant structural brain abnormalities, such as volumetric reductions in the hippocampus, anterior cingulate cortex and prefrontal cortex, as well as compromised white matter integrity. Recent growing evidence suggests that exercise is a promising and compelling treatment for depression in adults, showing effects that are comparable to other first-line treatments for depression.

**Objectives:**

This review aims to improve our understanding of the biological pathways involved in both the pathophysiology of depression and the antidepressant effects of exercise.

**Methods:**

This literature review considers the latest available scientific research addressing a comprehensive analysis of the antidepressant effect of physical exercise and the biological pathways involved.

**Results:**

Physical activity has been shown to have a multimodal effect that stimulates biochemical pathways and restores neuronal structures disturbed in depression. Experimental evidence supports exercise-induced increases in hippocampal, anterior cingulate cortex and prefrontal cortex volume, suggesting that exercise and antidepressant medication may alleviate depression through common neuromolecular mechanisms. However, the benefits of exercise may also persist beyond the end of treatment, unlike antidepressant medication.

**Conclusions:**

Given the undeniable scientific evidence favoring physical exercise in alleviating depression, it is of crucial importance to recommend this treatment in adjunct to psychotherapy and medication. Individuals at risk for depression also greatly benefit from it’s neuroprotective effects and should prioritize lifestyle changes. In older adults, there is a greater need for non-pharmaceutical treatments for depression due to limited efficacy of pharmaceutical treatments in this population.

